# Supplementary material for: LC-MS/MS Based Volatile Organic Compound Biomarkers Analysis for Early Detection of Lung Cancer
Source: Cancers (Basel). 2023 Feb 13;15(4):1186. doi: 10.3390/cancers15041186 (PMC9954752; doi:10.3390/cancers15041186)
Supplement: Supplementary file 1 [file cancers-15-01186-s001.zip › cancers-2164175-supplementary.pdf]

LC-MS/MS based volatile organic compound biomarkers analysis for early detection of lung cancer

Shuaibu Nazifi Sani[a], Wei Zhou[b], Balarabe B. Ismail[c], Yongkui Zhang[d], Zhijun Chen[d], Binjie Zhang[d], Changqian Bao[e], Houde Zhang[f]*, Xiaozhi Wang[a]*

[a]College of Information Science & Electronic Engineering, Zhejiang University, Hangzhou 310027, Zhejiang Province, China.

[b]Biochemical Analysis Lab.oratory, Breath (Hangzhou) Technology Co.,Ltd, Hangzhou 310000, Zhejiang Province, China.

[c]College of Biosystems Engineering and Food Science, Zhejiang University, Hangzhou 310058, China.

[d]Zhejiang Zhoushan Hospital, Zhou Shan 316021, Zhejiang Province, China.

[e]Department of Hematology, The Second Affiliated Hospital, College of Medicine ZhejiangUniversity,88# Jiefang Road Hangzhou 310009 Zhejiang Province, China.

[f]Department Gastroenterology, Nanshan Hapital, Guandong Medical University Shenzhen 518052, Guangdong province, China.

* Department of Information Science & Electronic Engineering, Zhejiang University,Hangzhou 310027, Zhejiang Province, China.

Corresponding author: Xiaozhi Wang (xw224@zju.edu.cn) and Houde Zhang (szkjk@126.com).

Supplementary Material

Introduction

Table S1. Potential biomarkers for lung cancer

Marker	CAS	Name	References
TG-1	78-93-3	2-Butanone	[1–5]
TG-2	123-72-8	Butyraldehyde，Butanal	[6]
TG-4	513-86-0	3-Hydroxy-2-butanone	[3,7–10]
TG-7	141-46-8	Glycolaldehyde	[3,10]
TG-8	107-87-9	2-Pentanone	[5,11]
TG-9	110-62-3	Valeraldehyde	[12–16]
TG-10	75-07-0	Acetaldehyde	[17–20]
TG-11	107-02-8	Acrolein	[21]
TG-12	98-001-01	2-Furaldehyde	[22,23]
TG-13	123-38-6	Propionaldehyde	[24]
TG-14	67-64-1	Acetone	[25]
TG-15	50-00-0	Formaldehyde	[19,26]
TG-16	66-25-1	Hexaldehyde	[4,15,27,28]
TG-17	111-71-7	Enanthaldehyde	[13,15,27,28]
TG-18	124-13-0	Octylaldehyde	[15,20,29]
TG-19	124-19-6	Nonaldehyde	[15,29,30]
TG-20	112-31-2	Decanal	[15,30]
TG-21	100-52-7	Benzaldehyde	[31–33]
TG-22	4170-30-3	Crotonaldehyde	[34]
TG-24	108-94-1	Cyclohexanone	[34]
TG-25	123-19-3	4-Heptanone	[35,36]

Material and Methods

The DNPH-coated silica gel solid-phase extraction column's preparation process

The DNPH reagent was dissolved in acetonitrile for three times of crystallization purification, and then prepared to 1000 mg/L with acetonitrile solution and 0.1% hydrochloric acid. The silica gel powder was washed with methanol 3 times before use, then dried in an oven at 80 °C overnight, and cooled for later use. 200 mg silica gel powder were added to a blank solid-phase extraction column for compaction, and 1000 mg/L DNPH solution was introduced under negative pressure to smear until saturation. Finally, the prepared solid-phase extraction column was dried under negative pressure, then sealed and refrigerated.

Factors influencing breath analysis

Environmental factors: during the breath collection process, the ambient air at the sampling site was collected for 6 months simultaneously.

Dietary factors (Table S5): breath samples were collected in a subgroup of 8 healthy non-smoker volunteers, and a dietary breath test was conducted as required to monitor the changes of VOC content in expiratory on an empty stomach and after different diets. The volunteers were asked to refrain from overeating and alcohol consumption two days before the test. After fasting for one night, the volunteers were given some common foods, including sugar-containing foods (bananas/bun/nuts), lipids (vegetable fat/tallow), proteins（egg white/milk), and others. Exhalation was collected on an empty stomach and at 5, 30, 60, 90, and 120 min after eating. After the common food experiment, it was found that sugar might have some influence on the exhaled air, so

the sugar drinking experiment was carried out (25 g glucose /25 g sucrose /10 g lactose /10 g xylose /10 mL lactulose), exhalation was also collected on an empty stomach and at 5, 30, 60, 90, and 120 min after drinking. Short-term protein and lipid diets do not affect exhaled breath, so long-term lipid and protein diet experiments (i.e., ketogenic diet) were carried out. 3 meals/day (meat: protein: carbohydrate = 7.5:2:0.5), the ketogenic diet lasts for 4 days, the normal diet is restored on the 5th and 6th day, and the exhaled breath is collected before each meal. Volunteers were required to rinse their mouths with water before each meal, and sample preparation was completed according to the same sample treatment scheme at the end of exhalation.

Exhalation method:8 healthy volunteers, after gargling with pure water and blowing in 2 Tadlar bags parallel to each other via the mouth and nasal cavity, respectively. Later, the subjects were given 5% glucose solution and asked to immediately exhale into Tedlar bags via the mouth and nasal cavity, respectively. Sample preparation was completed according to the same sample treatment scheme at the end of the exhalation collection.

Exhalation changes for the 30s with sugar in the mouth

The participants rinsed with pure water and then blew into two sampling bags in parallel as a control, then rinsed the mouth with 5% glucose solution for the 30s and then spat out. The exhalation was collected at 0, 3, 6, 9, 12, 15, 20, 25, 30, 40, 50, and 60 min, and the bags were prepared according to the same sample treatment scheme.

Table S2 MS conditions

	VOCs derivatives of DNPH	Q1 Mass (Da)	Q3 Mass (Da)	Retention Time (min)	DP (volts)	EP (volts)	CE (volts)	CXP (volts)
MRM (Negative ion mode)	TG-1-DNPH	251.05	152.05	11.02	-45	-15	-15	-15
		251.05	122	11.02	-45	-15	-15	-15
	TG-2-DNPH	250.9	152.05	11.28	-45	-15	-15	-15
		250.9	122	11.28	-45	-15	-15	-15
	TG-4-DNPH	267	152	6.14	-45	-15	-15	-15
		267	122	6.14	-45	-15	-15	-15
	TG-7-DNPH	239	122	3.86	-45	-15	-15	-15
		239	163	3.85	-45	-15	-15	-15

TG-8-DNPH	265.3	152	12.42	-45	-15	-15	-15
	265.3	122	12.42	-45	-15	-15	-15
TG-9-DNPH	265.05	152.05	12.61	-45	-15	-15	-15
	265.05	122	12.61	-45	-15	-15	-15
TG-10-DNPH	223.05	163.05	7.96	-60	-15	-15	-15
	223.05	122	7.96	-60	-15	-15	-15
TG-11-DNPH	234.95	158	9.24	-45	-15	-15	-15
	234.95	163.05	9.24	-45	-15	-15	-15
TG-12-DNPH	275.05	228.1	9.39	-45	-15	-15	-15
	275.05	181.1	9.39	-45	-15	-15	-15

TG-13-DNPH	237.05	163.1	9.69	-45	-15	-15	-15
	237.05	121.95	9.69	-45	-15	-15	-15
TG-15-DNPH	209	163.05	6.7	-45	-15	-15	-15
	209	151	6.7	-45	-15	-15	-15
TG-16-DNPH	279.1	152.05	13.77	-45	-15	-15	-15
	279.1	122	13.77	-45	-15	-15	-15
TG-17-DNPH	293	152.05	14.85	-45	-15	-15	-15
	293	122	14.85	-45	-15	-15	-15
TG-18-DNPH	307.15	152.05	15.79	-45	-15	-15	-15
	307.15	122	15.79	-45	-15	-15	-15

TG-19-DNPH	321.15	152.05	16.85	-45	-15	-15	-15
	321.15	122	16.85	-45	-15	-15	-15
TG-20-DNPH	335.15	152.05	17.87	-45	-15	-15	-15
	335.15	122	17.87	-45	-15	-15	-15
TG-21-DNPH	285	163.1	12	-45	-15	-15	-15
	285	121	12	-45	-15	-15	-15
TG-22-DNPH	248.95	172	10.6	-45	-15	-15	-15
	248.95	181.05	10.6	-45	-15	-15	-15
TG-24-DNPH	277	152	12.46	-45	-15	-15	-15
	277	122	12.47	-45	-15	-15	-15

	TG-25-DNPH	293.3	152	14.61	-45	-15	-15	-15
		293.3	122	14.6	-45	-15	-15	-15
Sectional scanning time	MRM detection window					60 s		
Cycling time	Target Cycle Time（across sMRexpts）					1.2 s		
Ion source parameter	Curtain Gas（CUR)					35		
	Collision Gas（CAD）					6		
	Ion Spray Voltage（IS）					-4500		
	Temperature（TEM）					500		
	Ion Source Gas 1（GS1）					50		
	Ion Source Gas 2（GS2）					50		

Table S3 Linear range, linear equation, correlation coefficient, LOD, LOQ and RSD of 21 VOCs

Compound	Linear range/(μg/L)	Linear equation	R	LOD/(μg/L)	LOQ/(μg/L)	RSD(n=6)/%
TG-1	0.75-30	Y=40493.9X-1423.5	0.999	0.25	0.75	0.59-1.56
TG-2	0.75-30	Y=46310.2X-1312.4	0.999	0.25	0.75	0.59-4.02
TG-4	0.8175-32.7	Y=31157.1X-1288.7	0.999	0.27	0.82	0.97-2.49
TG-7	0.25-10	Y=31432.6X+563.7	0.999	0.08	0.25	1.23-3.54
TG-8	0.74-29.6	Y=46739.0X-282.6	0.999	0.25	0.74	1.10-4.11
TG-9	0.75-30	Y=60027.6X-1706	0.999	0.25	0.75	0.96-1.52
TG-10	1-40	Y=41898.3X-1754	0.999	0.33	1.00	0.60-1.28
TG-11	0.75-30	Y=49316.1X+123.4	0.999	0.25	0.75	0.60-2.07
TG-12	0.75-30	Y=20596.7X-1700	0.999	0.25	0.75	1.30-4.21
TG-13	7.5-300	Y=69110.0X+56673.2	0.999	2.50	7.50	0.41-1.40
TG-14	50-5000	Y=9125310.0X+237633	0.999	16.67	50.00	1.00-5.00
TG-15	7.5-300	Y=34908.7X+76576.4	0.998	2.50	7.50	1.43-4.46
TG-16	0.75-30	Y=66208.4X-1237.6	0.999	0.25	0.75	0.54-1.70
TG-17	0.75-30	Y=61668.1X-480.5	0.999	0.25	0.75	1.04-1.99

TG-18	0.75-30	Y=50573.7X-319.2	0.999	0.25	0.75	0.58-1.83
TG-19	1.5-60	Y=43149.9X+1880.6	0.999	0.50	1.50	0.71-1.86
TG-20	1.5-60	Y=22323.0X+4916.6	0.999	0.50	1.50	0.73-4.85
TG-21	0.75-30	Y=42739.5X+830.9	0.999	0.25	0.75	1.11-2.96
TG-22	0.75-30	Y=46149.5X-1839.5	0.999	0.25	0.75	0.50-1.81
TG-24	0.1-3	Y=6083.4X+115.3	0.999	0.03	0.10	2.31-4.72
TG-25	0.194-7.76	Y=66642.7X+2675.9	0.999	0.06	0.19	0.52-3.85

Results and Discussion

Table S4. The AUC for the ROC curves to discriminate AIS/MIA/IAC of early lung cancer and control group

AUC	Healthy controls	Early lung cancer		
		AIS	MIA	IAC
TG-4	0.766	/	/	0.796
TG-7	0.611	0.637	0.606	0.627
TG-8	0.636	/	/	/
TG-11	0.682	0.645	0.703	0.727
TG-13	/	/	0.658	0.690
TG-19	0.604	0.624	/	0.655
TG-20	0.611	0.533	0.617	0.680
TG-22	0.625	0.658	0.641	0.637

Table S5. Different foods and intake quantity

Classification		Name	Intake
		Nuts	100 g
	Sugar-containing foods	Bun	100 g
		Bananas	100 g
Common foods		Milk	500 mL
	Proteins	Egg white	50 g
		Vegetable fat	20 mL
	Lipids	Tallow	20 g
	Other	Alcohol	50 mL
		Glucose	25 g
		Sucrose	25 g
Sugar		Nuts	10 g
		Bun	10 g
		Bananas	10 mL

Table S6. The level of TG-4 after the intake of different sugars.

Sugar	Glycemic index (GI)	TG-4 concentration at 5 min（μg/L）	Intake
Glucose	100	81.97	25 g
Sucrose	65	33.55	25 g
Lactose	46	6.5	10 g
Xylose	17.2	4.34	10 g
Lactulose	5	2.5	10 mL

Pearson correlation (r = 0.975)

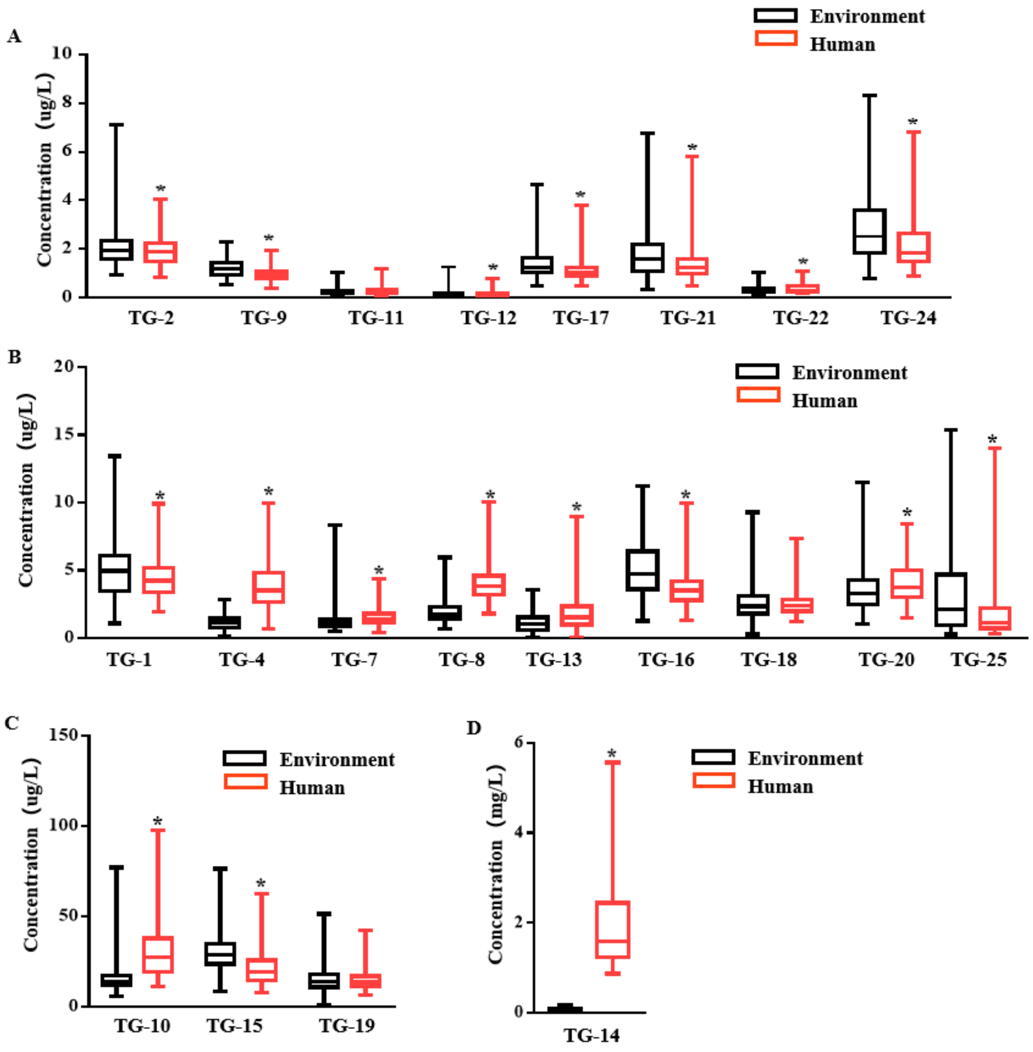

Figure S1. Differences in the concentration of VOCs in the environment and human breath. A-D. Among the 21 VOCs, there are 10 VOCs in the environment that were higher than the human exhaled breath, the concentration of the remaining 11 VOCs in the environment is similar to or lower than that of human exhaled breath. Values and error bars represent mean ± SE. *, $P < 0.05$.

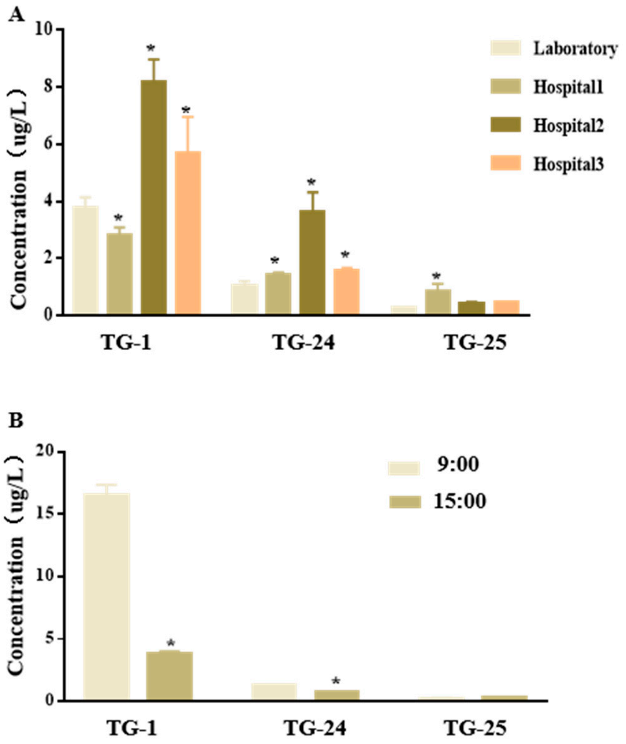

Figure S2. VOCs concentrations at different locations and at different times. A. Ambient air was collected multiple times at the same location in different hospitals. Compared with the laboratory environment, TG-1, TG-24, and TG-25 showed different concentrations. B. The ambient air was collected at the same location in the laboratory at 9:00 and 15:00, and different VOCs in the ambient air also showed different concentrations. Values and error bars represent mean ± SE. * means compared with laboratory, P<0.05.

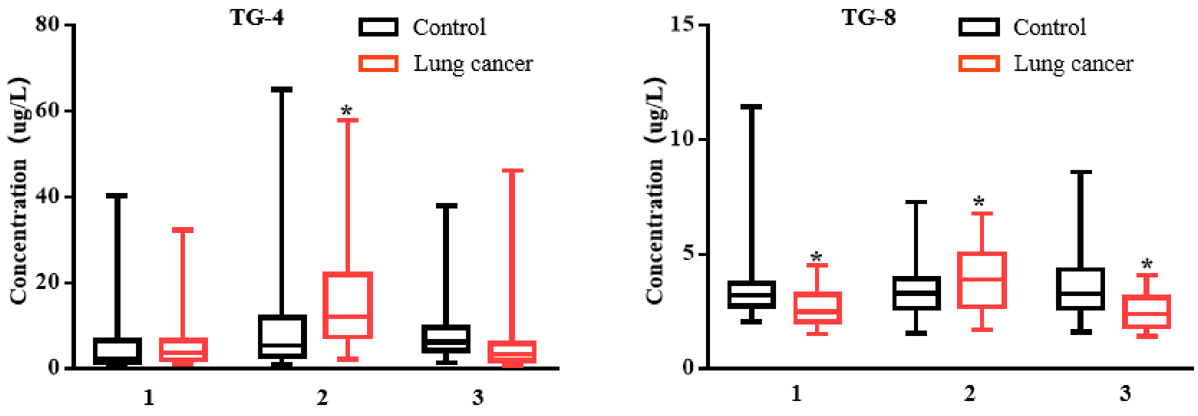

Figure S3. The preliminary experiment comparing the concentration of VOCs in the exhaled breath between the control group and the lung cancer group. 1, fasting;2, 1h after the meal;3, 2 h after the meal; Among the 11 carbonyls VOCs, only TG-4 and TG-8 are different. Compared with the control group, TG-4 in the exhaled breath of lung cancer patients is higher at 1 hour after a meal, and TG-8 is higher at 3-time points. *, $P < 0.05$.

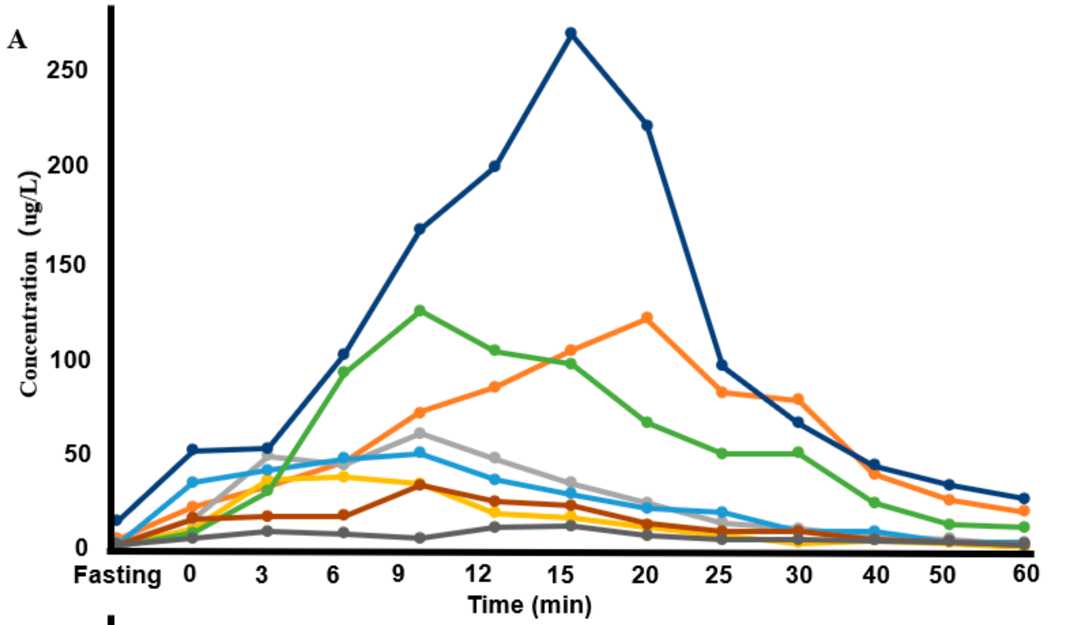

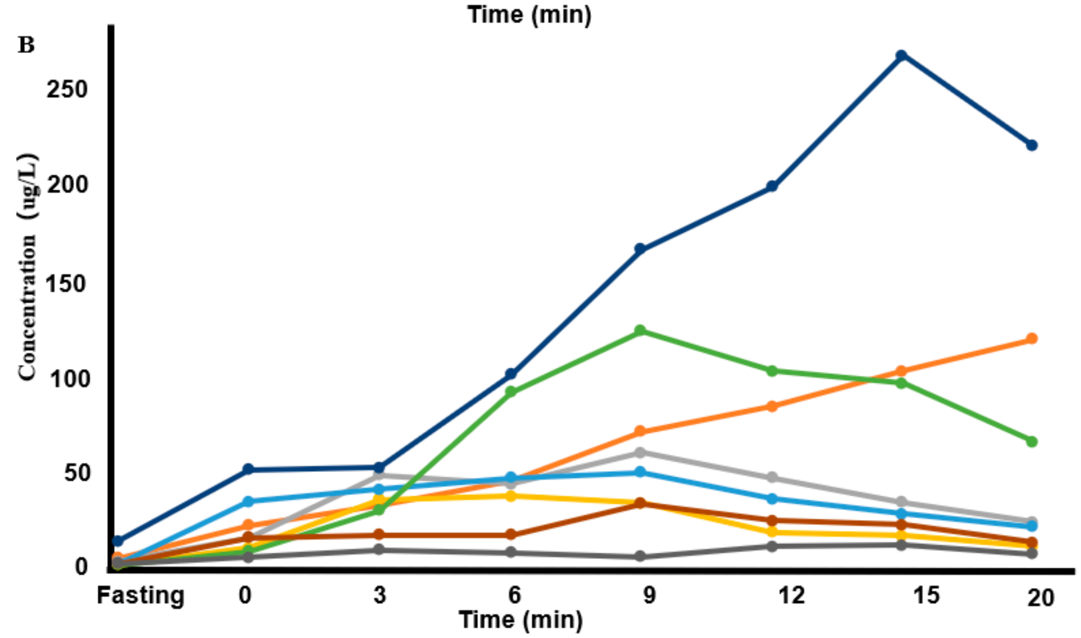

Figure S4. Changes in the concentration of TG-4 in exhaled breath after sugar in the mouth for the 30 s, the color indicates TG-4 concentration pattern of 8 healthy volunteers. A. Changes in the concentration of TG-4 in the oral exhaled breath of 8 healthy volunteers after sugar in the mouth for the 30 s. B. The concentration of TG-4 in the exhaled breath of 8 healthy volunteers reached the highest value within 20 minutes after sugar in the mouth for the 30 s.

Table S7. VOCs Statistical data for control, Lung cancer before the operation, Lung cancer three to seven days after surgery , and Lung cancer four to six weeks after surgery

VOCs	Groups	Statistical data	
		Mean	SD
TG-4	Control	6.08	6.10
	Lung cancer before surgery	19.48	24.63
	Lung cancer 3-7 days after surgery	12.16	17.66
	Lung cancer 4-6 weeks after surgery	11.12	13.52
TG-7	Control	1.70	0.91
	Lung cancer before surgery	1.24	0.46
	Lung cancer 3-7 days after surgery	1.12	0.32
	Lung cancer 4-6 weeks after surgery	1.10	0.36
TG-8	Control	3.30	1.55
	Lung cancer before surgery	4.38	2.12
	Lung cancer 3-7 days after surgery	2.81	1.16
	Lung cancer 4-6 weeks after surgery	3.52	1.04
TG-11	Control	0.39	0.20
	Lung cancer before surgery	0.25	0.17
	Lung cancer 3-7 days after surgery	0.28	0.13
	Lung cancer 4-6 weeks after surgery	0.33	0.26
TG-19	Control	15.46	5.93
	Lung cancer before surgery	14.27	4.89
	Lung cancer 3-7 days after surgery	11.43	5.27
	Lung cancer 4-6 weeks after surgery	8.27	3.29
TG-20	Control	4.81	2.09
	Lung cancer before surgery	4.86	1.86
	Lung cancer 3-7 days after surgery	3.89	1.43
	Lung cancer 4-6 weeks after surgery	3.17	1.24
TG-22	Control	0.38	0.18
	Lung cancer before surgery	0.42	0.10
	Lung cancer 3-7 days after surgery	0.32	0.15
	Lung cancer 4-6 weeks after surgery	0.23	0.09

References

[1] S.M. Gordon; J.P. Szldon, B.K. Krotoszynski; R.D. Gibbons; H.J.O.’ Neill. VolatileOrganicCompoundsin ExhaledAir from Patientswith LungCancer Sampling and Analysis, Clin. Chem. Cunical Chem. 318 (1985) 1278–1282.

[2] B. Buszewski; A. Ulanowska; T. Kowalkowski; K. Cieliski. Investigation of lung cancer biomarkers by hyphenated separation techniques and chemometrics, Clin. Chem. Lab. Med. 50 (2012) 573–581. https://doi.org/10.1515/cclm.2011.769.

[3] X.-A. Fu; M. Li, R.J. Knipp; M.H. Nantz; M. Bousamra. Noninvasive detection of lung cancer using exhaled breath, Cancer Med. 3 (2014) 174–181. https://doi.org/10.1002/cam4.162.

[4] Y. Saalberg; M. Wolff. VOC breath biomarkers in lung cancer, Clin. Chim. Acta. 459 (2016) 5–9. https://doi.org/10.1016/j.cca.2016.05.013.

[5] R. Fernández del Río; M.E. O’Hara; A. Holt; P. Pemberton; T. Shah; T. Whitehouse; C.A. Mayhew. Volatile Biomarkers in Breath Associated With Liver Cirrhosis - Comparisons of Pre- and Post-liver Transplant Breath Samples, EBioMedicine. 2 (2015) 1243–1250. https://doi.org/10.1016/j.ebiom.2015.07.027.

[6] W. Song2006ready. Identification of bacteria using volatile organic compounds, Phil. Mag. 27 (2017) 63(2):112–21.

[7] I. Nardi-Agmon; M. Abud-Hawa; O. Liran; N. Gai-Mor; M. Ilouze; A. Onn; J. Bar; D. Shlomi; H. Haick; N. Peled. Exhaled breath analysis for monitoring response to treatment in advanced lung cancer, J. Thorac. Oncol. 11 (2016) 827–837. https://doi.org/10.1016/j.jtho.2016.02.017.

[8] T. Itoh; T. Akamatsu; A. Tsuruta; W. Shin. Selective detection of target volatile organic compounds in contaminated humid air using a sensor array with principal component analysis, Sensors (Switzerland). 17 (2017). https://doi.org/10.3390/s17071662.

[9] Y.I. Yamada; G. Yamada; M. Otsuka; H. Nishikiori; K. Ikeda; Y. Umeda; H. Ohnishi; K. Kuronuma; H. Chiba; J.I. Baumbach; et al. Volatile Organic Compounds in Exhaled Breath of Idiopathic Pulmonary Fibrosis for Discrimination from Healthy Subjects, Lung. 195 (2017) 247–254. https://doi.org/10.1007/s00408-017-9979-3.

[10] E.M. Schumer; J.R. Trivedi; V. Van Berkel; M.C. Black; M. Li; X.A. Fu; M. Bousamra. High sensitivity for lung cancer detection using analysis of exhaled carbonyl compounds Read at the 95th Annual Meeting of the American Association for Thoracic Surgery, Seattle, Washington, April 25-29, 2015., J. Thorac. Cardiovasc. Surg. 150 (2015) 1517–1524. https://doi.org/10.1016/j.jtcvs.2015.08.092.

[11] P.M. Santos; M. del Nogal Sánchez; Á.P.C. Pozas; J.L.P. Pavón; B.M. Cordero. Determination of ketones and ethyl acetate—a preliminary study for the discrimination of patients with lung cancer, Anal. Bioanal. Chem. 409 (2017) 5689–5696. https://doi.org/10.1007/s00216-017-0508-2.

[12] Z. Li; C. Xu; J. Shu; B. Yang; Y. Zou. Doping-assisted low-pressure photoionization mass spectrometry for the real-time detection of lung cancer-related volatile organic compounds, Talanta. 165 (2017) 98–106. https://doi.org/10.1016/j.talanta.2016.12.039.

[13] J. Obermeier; P. Trefz; J. Happ; J.K. Schubert; H. Staude; D.C. Fischer; W. Miekisch. Exhaled volatile substances mirror clinical conditions in pediatric chronic kidney disease http://journals.plos.org/plosone/article/file?id=10.1371/journal.pone.0178745&type=printable, PLoS One. 12 (2017). https://doi.org/10.1371/journal.pone.0178745.

[14] P. Trefz; J. Obermeier; R. Lehbrink; J.K. Schubert; W. Miekisch; D.C. Fischer. Exhaled volatile substances in children suffering from type 1 diabetes mellitus: results from a cross-sectional study, Sci. Rep. 9 (2019) 1–9. https://doi.org/10.1038/s41598-019-52165-x.

[15] S. Kumar; J. Huang; N. Abbassi-Ghadi; H.A. MacKenzie; K.A. Veselkov; J.M. Hoare; L.B. Lovat; P. Spanel; D. Smith; G.B. Hanna; et al. Mass spectrometric analysis of exhaled breath for the identification of volatile organic compound biomarkers in esophageal and gastric adenocarcinoma, Ann. Surg. 262 (2015) 981–990. https://doi.org/10.1097/SLA.0000000000001101.

[16] D. Poli; M. Goldoni; M. Corradi; O. Acampa; P. Carbognani; E. Internullo; A. Casalini; A. Mutti. Determination of aldehydes in exhaled breath of patients with lung cancer by means of on-fiber-derivatisation SPME-GC/MS, J. Chromatogr. B Anal. Technol. Biomed. Life Sci. 878 (2010) 2643–2651. https://doi.org/10.1016/j.jchromb.2010.01.022.

[17] K. Schallschmidt; R. Becker; C. Jung; W. Bremser; T. Walles; J. Neudecker; G. Leschber; S. Frese; I. Nehls. Comparison of volatile organic compounds from lung cancer patients and healthy controls—challenges and

limitations of an observational study, J. Breath Res. 10 (2016) 046007. https://doi.org/10.1088/1752-7155/10/4/046007.

[18] J. Huang; S. Kumar; N. Abbassi-Ghadi; P. Španěl; D. Smith; G.B. Hanna. Selected ion flow tube mass spectrometry analysis of volatile metabolites in urine headspace for the profiling of gastro-esophageal cancer, Anal. Chem. 85 (2013) 3409–3416. https://doi.org/10.1021/ac4000656.

[19] S. Kumar; J. Huang; J.R. Cushnir; P. Španěl; D. Smith; G.B. Hanna. Selected ion flow tube-MS analysis of headspace vapor from gastric content for the diagnosis of gastro-esophageal cancer, Anal. Chem. 84 (2012) 9550–9557. https://doi.org/10.1021/ac302409a.

[20] Y. Wei; M. Wang; H. Liu; Y. Niu; S. Wang; F. Zhang; H. Liu. Simultaneous determination of seven endogenous aldehydes in human blood by headspace gas chromatography–mass spectrometry, J. Chromatogr. B Anal. Technol. Biomed. Life Sci. 1118–1119 (2019) 85–92. https://doi.org/10.1016/j.jchromb.2019.04.027.

[21] P. Sarkar. Response of DNA damage genes in acrolein-treated lung adenocarcinoma cells, Mol. Cell. Biochem. 450 (2019) 187–198. https://doi.org/10.1007/s11010-018-3385-x.

[22] C. Silva; R. Perestrelo; P. Silva; F. Capelinha; H. Tomás; J.S. Câmara. Volatomic pattern of breast cancer and cancer-free tissues as a powerful strategy to identify potential biomarkers, Analyst. 144 (2019) 4153–4161. https://doi.org/10.1039/c9an00263d.

[23] Z.Q. Xu; Y.Y. Broza; R. Ionsecu; U. Tisch; L. Ding; H. Liu; Q. Song; Y.Y. Pan; F.X. Xiong; K.S. Gu; et al. A nanomaterial-based breath test for distinguishing gastric cancer from benign gastric conditions, Br. J. Cancer. 108 (2013) 941–950. https://doi.org/10.1038/bjc.2013.44.

[24] A. Ulanowska; T. Kowalkowski; E. Trawińska; B. Buszewski. The application of statistical methods using VOCs to identify patients with lung cancer, J. Breath Res. 5 (2011) 046008. https://doi.org/10.1088/1752-7155/5/4/046008.

[25] Y. Hanai; K. Shimono; K. Matsumura; A. Vachani; S. Albelda; K. Yamazaki; G.K. Beauchamp; H. Oka. Urinary volatile compounds as biomarkers for lung cancer, Biosci. Biotechnol. Biochem. 76 (2012) 679–684. https://doi.org/10.1271/bbb.110760.

[26] Z. Tong; W. Wang; W. Luo; J. Lv; H. Li; H. Luo; J. Jia; R. He. Urine formaldehyde predicts cognitive impairment in post-stroke dementia and Alzheimer's disease, J. Alzheimer's Dis. 55 (2017) 1031–1038. https://doi.org/10.3233/JAD-160357.

[27] A.L. Oenning; L. Morés; A.N. Dias; E. Carasek. A new configuration for bar adsorptive microextraction (BAμE) for the quantification of biomarkers (hexanal and heptanal) in human urine by HPLC providing an alternative for early lung cancer diagnosis, Anal. Chim. Acta. 965 (2017) 54–62. https://doi.org/10.1016/j.aca.2017.02.034.

[28] J.F. Liu; B.F. Yuan; Y.Q. Feng. Determination of hexanal and heptanal in human urine using magnetic solid phase extraction coupled with in-situ derivatization by high performance liquid chromatography, Talanta. 136 (2015) 54–59. https://doi.org/10.1016/j.talanta.2015.01.003.

[29] J. Li; Y. Peng; Y. Liu; W. Li; Y. Jin; Z. Tang; Y. Duan. Investigation of potential breath biomarkers for the early diagnosis of breast cancer using gas chromatography-mass spectrometry, Clin. Chim. Acta. 436 (2014) 59–67. https://doi.org/10.1016/j.cca.2014.04.030.

[30] P. Fuchs; C. Loeseken; J.K. Schubert; W. Miekisch. Breath gas aldehydes as biomarkers of lung cancer, Int. J. Cancer. 126 (2010) 2663–2670. https://doi.org/10.1002/ijc.24970.

[31] Z. Jia; H. Zhang; C.N. Ong; A. Patra; Y. Lu; C.T. Lim; T. Venkatesan. Detection of Lung Cancer: Concomitant Volatile Organic Compounds and Metabolomic Profiling of Six Cancer Cell Lines of Different Histological Origins, ACS Omega. 3 (2018) 5131–5140. https://doi.org/10.1021/acsomega.7b02035.

[32] H. Haick; Y.Y. Broza; P. Mochalski; V. Ruzsanyi; A. Amann. Assessment, origin, and implementation of breath volatile cancer markers, Chem. Soc. Rev. 43 (2014) 1423–1449. https://doi.org/10.1039/c3cs60329f.

[33] M. Bouza; J. Gonzalez-Soto; R. Pereiro; J.C. De Vicente; A. Sanz-Medel. Exhaled breath and oral cavity VOCs as potential biomarkers in oral cancer patients, J. Breath Res. 11 (2017). https://doi.org/10.1088/1752-7163/aa5e76.

[34] S.L. Park; S.G. Carmella; M. Chen; Y. Patel; D.O. Stram; C.A. Haiman; L. Le Marchand; S.S. Hecht. Mercapturic acids derived from the toxicants acrolein and crotonaldehyde in the urine of cigarette smokers from five ethnic groups with differing risks for lung cancer, PLoS One. 10 (2015) 1–17. https://doi.org/10.1371/journal.pone.0124841.

[35] I. Kohl; J. Beauchamp; F. Cakar-Beck; J. Herbig; J. Dunkl; O. Tietje; M. Tiefenthaler; C. Boesmueller; A. Wisthaler; M. Breitenlechner; et al. First observation of a potential non-invasive breath gas biomarker for kidney function, J. Breath Res. 7 (2013) 017110. https://doi.org/10.1088/1752-7155/7/1/017110.

[36] pone.0046258 1..9 _ Enhanced Reader.pdf, (n.d.).
